# Exploring Anti-Neoplastic Activity of Chitosan Nanobubbles Decorated with ICOS-Fc and Loaded with Paclitaxel in a Human and Murine Model of Melanoma

**DOI:** 10.3390/pharmaceutics17121530

**Published:** 2025-11-28

**Authors:** Deepika Pantham, Monica Argenziano, Foteini Christaki, Nausicaa Clemente, Chiara Colombo, Elisa Benetti, Stefania Pizzimenti, Umberto Dianzani, Ian Stoppa, Roberta Cavalli, Chiara Dianzani

**Affiliations:** 1 Department of Health Sciences, Interdisciplinary Research Center of Autoimmune Diseases-IRCAD, University of Piemonte Orientale, 28100 Novara, Italy; deepika.pantham@uniupo.it (D.P.); foteini.christaki@uniupo.it (F.C.); nausicaa.clemente@uniupo.it (N.C.); 20043170@studenti.uniupo.it (C.C.); umberto.dianzani@uniupo.it (U.D.); ian.stoppa@uniupo.it (I.S.); 2 Department of Scienza e Tecnologia del Farmaco, University of Torino, 10125 Torino, Italy; monica.argenziano@unito.it (M.A.); elisa.benetti@unito.it (E.B.); chiara.dianzani@unito.it (C.D.); 3 Department of Clinical and Biological Sciences, University of Torino, 10125 Torino, Italy

**Keywords:** paclitaxel, melanoma, angiogenesis, tumor growth, D4M-3A melanoma model, nanobubbles, ICOS-Fc, co-delivery

## Abstract

**Background:** Paclitaxel (PTX) is an anti-neoplastic drug that inhibits not only melanoma cell proliferation but also migration and angiogenesis. ICOS-Fc is a recombinant molecule that triggers ICOS ligand (ICOSL) on tumor cells and cells of the tumor microenvironment and inhibits tumor growth, angiogenesis, and metastasis. This study investigated the effects of chitosan nanobubbles loaded with low doses of PTX and surface decorated with ICOS-Fc (ICOS-Fc-NB-PTX) in inhibiting in vitro and in vivo melanoma cell growth and invasiveness. **Methods:** Preparation and characterization of nanoformulations, as well as in vitro drug release studies, were carried out. Nanoformulations were studied both in vitro and in vivo. In melanoma cells, viability, migration, and invasion assays were analyzed. For the in vivo experiments, C57BL/6 Wild-type (WT) male mice were injected subcutaneously with D4M-3A cells, a murine melanoma cell line engineered to carry the BRAF^V600E^ mutation. After treatments, in vivo tumor growth, proliferation, and angiogenesis markers were studied. **Results**: In vitro tests showed the great ability of ICOS-Fc-NB-PTX to inhibit cell viability, migration, and invasion. These results were confirmed in vivo, where the tumors of mice treated with ICOS-Fc-NB-PTX displayed decreased growth accompanied by downregulation of the proliferation marker Ki-67 and reduced development of CD31^+^ blood vessels. **Conclusions**: In conclusion, the ICOS-Fc-NB-PTX formulation deserves to be further analyzed as a highly effective combination for melanoma, exerting multifaceted anti-tumor activities.

## 1. Introduction

Melanoma is the most aggressive and invasive malignant neoplasm affecting the skin. It accounts for only 5% of total skin cancers, but it causes about three-quarters of the deaths from skin tumors [1]. Its incidence has substantially increased in the last 50 years and almost doubled in the last decade [1,2,3].

Early surgery is the only effective approach to eradicate melanoma, but often, based on the staging and mutational state, it must be accompanied by radiotherapy and therapy with anti-neoplastic drugs, which include conventional chemotherapeutics (e.g., dacarbazine, temozolomide, paclitaxel, cisplatin, and carboplatin) showing low efficacy, targeted therapies that are effective only on tumors carrying selected mutations, and immunotherapy using immune checkpoint inhibitors capable of increasing the anti-tumor immune response by counteracting the tumor-mediated immune suppression [4,5].

To date, the Food and Drug Administration (FDA) has approved immune checkpoint inhibitors targeting either cytotoxic T-lymphocyte antigen-4 (CTLA-4), counteracting the activity of regulatory T (Treg) cells, or programmed cell death protein-1 (PD-1) and its ligand (PDL-1), counteracting the exhaustion of effector T cells [6]. However, the rate of non-responder patients is high, and novel, valuable alternatives are needed, including combinations of conventional chemotherapies and immunotherapies. In this light, paclitaxel (PTX) is a promising drug because it induces direct cytotoxicity of tumor cells by interfering with microtubule function, causing an immunogenic death and promoting the activation of anti-tumor cytotoxic T cells [7], which might be further boosted by immune checkpoint inhibitors. However, the use of PTX is limited by its nonselective distribution and poor water solubility, which necessitates the use of solvents and causes substantial side effects. These limitations may be overcome by loading PTX in nanocarriers, improving its distribution and stability, and decreasing adverse effects such as nephrotoxicity and neurotoxicity [8]. Abraxane^®^, consisting of PTX bound to human albumin nanoparticles, Genexol-PM^®^, polymeric micelles, and Lipusu^®^, a liposomal formulation, are marketed PTX medical products that showed an increased therapeutic index of the drug and tumor accumulation by the EPR effect. In this context, much research attention was focused on the design of polymeric and lipidic nanodelivery systems for PTX administration [8].

Previously, we showed that PTX loaded in pyromellitic nanosponges is highly effective in inhibiting not only the growth and migration of melanoma cell lines in vitro, but also the in vivo growth of established B16 melanoma tumors in mice, where it exerts substantial anti-proliferative and anti-angiogenic effects [9]. Interestingly, co-delivery strategies have been investigated to further increase the efficacy of PTX [10,11]. In particular, its combination with immunotherapeutic agents may produce a synergistic effect, enhancing anti-tumor immune response [12]. Inducible Co-Stimulator (ICOS) is a costimulatory receptor of T cells, belonging to the CD28 family, which also includes CTLA-4 and PD-1. ICOS is expressed on activated T cells and binds ICOS ligand (ICOSL) expressed on several types of immune and non-immune cells, including endothelial cells, epithelial cells, fibroblasts, and many types of cancer cells [13].

ICOS triggering by ICOSL induces costimulatory signals that enhance the activation and effector functions of T cells, but it is also involved in the development of highly active regulatory T (Treg) cells [14,15]. On the other side, the triggering of ICOSL by ICOS transduces a “reverse signal” modulating several activities of the ICOSL-expressing cell. A key effect of this reverse signaling is on cell migration, which is inhibited in dendritic cells, endothelial cells, and several types of tumor cells. Moreover, in dendritic cells, it promotes antigen cross-presentation in class I MHC molecules, favors the activation of cytotoxic T cells, and increases the secretion of IL-10 and IL-23, promoting the development of Treg and type 17 T helper (Th17) cells, respectively [16,17]. In tumor cells, it also suppresses the epithelial-to-mesenchymal transition (EMT), which is involved in tumor malignancy [16,17]. These effects can be elicited in vitro by ICOSL triggering using the recombinant protein ICOS-Fc, which combines the extracellular portion of ICOS with the Fc portion of IgG1.

ICOS-Fc has been used also in several in vivo setting in mice showing that treatment with free ICOS-Fc inhibits development of osteoporosis by inhibiting osteoclast activity, decreases severity of sepsis by inhibiting inflammation and organ injury, favors liver repair in acute damage induced by CCl4 by supporting development of M2 type macrophages, favors skin wound healing by supporting angiogenesis and development of M2 macrophages, and inhibits metastatic diffusion into the lungs of several types of tumor cell lines injected in the mouse tail vein. By contrast, free ICOS-Fc has no effects on the growth of established melanomas in vivo, but it displays a strong anti-tumor activity when it is delivered upon encapsulation in nanoparticles, favoring its accumulation in the tumor mass due to the enhanced permeability and retention (EPR) effect [18,19,20]. In particular, ICOS-Fc loaded in β-cyclodextrin nanosponges (CDNS) or poly(lactic-co-glycolic) acid nanoparticles (PLGA) inhibits the growth of established B16-F10 melanomas by suppressing tumor angiogenesis and, in part, the development of regulatory cells [21]. Moreover, the anti-tumor effect of ICOS-Fc can cooperate with other anti-neoplastic drugs since lipid nanoemulsions co-encapsulating ICOS-Fc, sorafenib, and temozolomide show higher anti-tumor activity in vivo against the B16-F10 melanoma compared to the same nanoemulsions loaded with sorafenib plus temozolomide or with ICOS-Fc alone [22].

Nanobubbles (NBs) are biocompatible and biodegradable nanoparticles characterized by a gas-filled “core shell” structure. The external structure, the shell, can be composed of proteins, lipids, or polymers, and the core can be filled with different gases or vaporizable compounds, such as perfluorocarbons [23]. Chitosan, a shellfish-derived biomaterial, can be preferred as an essential component of the shell of the NB, thanks to its biocompatibility, low immunogenicity, low toxicity, and antibacterial activities [24,25]. Thanks to the different domains present in NB nanostructures with their own polarity, they are able to transport with high payload both hydrophobic and hydrophilic molecules [26]. In addition, this nanostructure is suitable for the co-delivery of drugs and macromolecules in the same system.

The aim of this work was to investigate chitosan-shelled NBs for the co-delivery of low doses of PTX and ICOS-Fc into the tumor mass. The use of chitosan NBs was suggested for its known efficacy in exploiting the EPR effect to deliver anticancer drugs to the tumor site [27,28], and by its capability of co-delivering different molecules. To the best of our knowledge, ICOS-Fc conjugated NBs are described here for the first time. The combination of PTX and ICOS-Fc delivered by NBs can represent a novel chemo-immunotherapy strategy for improving melanoma treatment outcomes.

The capability of ICOS-Fc-NB-PTX to inhibit the proliferation and migration of different melanoma cell lines in vitro and to decrease the in vivo growth of established subcutaneous D4M-3A melanomas bearing the BRAF^V600E^ mutation in mice has been investigated. The chosen animal species and model were selected because they closely replicate the biological processes relevant to investigating the effects of NB-delivered ICOS-Fc, PTX, and their combination on melanoma growth in a controlled and reproducible manner.

## 2. Materials and Methods

### 2.1. Reagents and Antibodies

PTX, Crystal Violet, phosphate-buffered saline (PBS), formalin, H_2_O_2_, Triton X-100, dimethyl sulfoxide (DMSO), 3-(4,5-dimethyl thiazol-2-yl)-2,5-diphenyltetrazolium bromide (MTT), and hematoxylin were from Merk Life Science S.r.l. (Milan, Italy). RPMI1640 medium, Dulbecco’s Modified Eagle Medium (DMEM) high glucose, fetal bovine serum (FBS), penicillin, streptomycin, and trypsin were from Euroclone (Pero, Milan, Italy). Transwell Boyden chambers and Matrigel were from BD Biosciences (Milan, Italy). ICOS-Fc was purchased from Novaicos ImmunoTherapeutics (Novara, Italy). Normal goat serum was obtained by R&D Systems (Minneapolis, MN, USA). Citrate buffer was purchased from Vector Laboratories (Burlingame, CA, USA). Polyclonal rabbit anti-CD31 and monoclonal rabbit anti-Ki-67 antibodies were from Abcam (Cambridge, UK). The anti-rabbit-HRP produced in goat was purchased from Thermo Fisher Scientific (Waltham, MA, USA). 3,30-diaminobenzidine (DAB) was obtained from Agilent Dako (Santa Clara, CA, USA).

Unless otherwise stated, all the materials used were purchased from Sigma-Aldrich, Merck KGaA (Darmstadt, Germany). Epikuron 200^®^ was kindly supplied by Cargill (Hamburg, Germany).

### 2.2. Preparation of NB Nanoformulations Loaded with PTX and ICOS-Fc

Blank NBs were formulated using chitosan with a low molecular weight (average MW 100,000 Da, degree of deacetylation 75–85%) and decafluoropentane as the shell and core components, respectively. NBs were prepared according to a tuned multi-step protocol, previously described [29]. Briefly, a nanoemulsion was obtained by adding an ethanol solution of Epikuron 200 (1% *w*/*v*) and palmitic acid (1% *w*/*v*) to decafluoropentane and ultrapure water, followed by homogenization with an UltraTurrax SG215 homogenizer (IKA, Konigswinter, Germany) at 25,000 rpm for 3 min. Then, for the production of the chitosan shell, a chitosan solution (2% *w*/*v*, pH 5.0) was added dropwise to the preformed nanoemulsion. Fluorescent NBs and ICOS-Fc-NBs were obtained by adding 6-coumarin to the decafluoropentane (0.1% *w*/*v*). PTX-NBs were prepared by dissolving the drug in the decafluoropentane using isopropanol as a co-solvent and following the same preparation protocol described above. A purification step by dialysis (cellulose membrane Spectra/Pore, cutoff 12,000 Da) was conducted to eliminate the unloaded drug. The PTX concentration in the NB was determined by high-performance liquid chromatography (HPLC) analysis using a Perkin Elmer pump (Perkin Elmer PUMP 250B) equipped with a UV/Vis spectrophotometer detector (Flexar UV/Vis LC spectrophotometer detector, Perkin Elmer, Waltham, MA, USA) set at the wavelength of 227 nm. A reverse phase Agilent TC C18 column was used (150 cm × 4.6 mm, pore size 5 μm (Agilent Technologies, Santa Clara, CA, USA)), eluted with a mobile phase composed of a mixture of acetonitrile and water (60:40 *v*/*v*) at a flow rate of 1 mL/min.

ICOS-Fc decorated NBs were obtained by the conjugation of ICOS-Fc (100 μg/mL) to the NB chitosan shell through EDC-NHS chemistry. Carboxylic groups from ICOS-Fc were activated with 1-Ethyl-3-(3-dimethylaminopropyl)carbodiimide (EDC) and N-Hydroxysuccinimide (NHS), followed by amide bond conjugation with the amino groups of chitosan present on the NB surface. Briefly, 100 μg of ICOS-Fc was dissolved in 100 μL PBS, and 25 μL of a solution of EDC (0.4 mg/mL) and NHS (0.6 mg/mL) was added to the sample and kept under magnetic stirring for 15 min at room temperature. The activated ICOS-Fc solution was then added to 1 mL of chitosan NBs, and the reaction was carried out for 2 h under stirring [30]. For ICOS-Fc quantification, the Pierce™ BCA Protein Assay Kit (Thermo Scientific, Rockford, IL, USA) was used.

### 2.3. Characterization of NB Nanoformulations

NB formulations (blank, fluorescent, loaded with PTX, and decorated with ICOS-Fc) were characterized by dynamic light scattering (DLS) using a 90 Plus Instrument (Brookhaven Instrument Co., Holtsville, NY, USA, fixed scattering angle of 90°) to determine their physico-chemical parameters (average diameter, polydispersity index, and zeta potential). The samples were diluted in distilled water (1:30 *v*/*v*) before analysis. The results are reported as the average of three measurements carried out after independent experiments.

The loading capacity was evaluated on freeze-dried NB samples. Briefly, a weighted amount of freeze-dried PTX-loaded NBs was dispersed in 5 mL of isopropanol, and, after sonication and centrifugation, the supernatant was analyzed by HPLC. The loading capacity was calculated according to the equation: (amount of PTX loaded in NB/weight of freeze-dried NB) × 100. The encapsulation efficiency was determined using the equation: (amount of PTX loaded in NB/total amount of PTX) × 100.

The physical stability of NB formulations stored at 4 °C was evaluated by measuring the physico-chemical parameters and PTX concentration over time up to 6 months.

The presence of ICOS-Fc on the surface of the NBs was assessed by ELISA. Briefly, recombinant ICOSL-HIS (Sino Biological Europe GmbH, Eschborn, Germany) was immobilized on the bottom of a Nunc MaxiSorp 96-well plate (Waltham, MA, USA) by overnight incubation at 4 °C. The following day, wells were washed with PBS containing 0.05% Tween-20 and blocked with 5% non-fat dry milk in PBS for 1 h at room temperature.

ICOS-Fc-conjugated NBs at a concentration of 100 µg/mL were tested at serial dilutions ranging from 1:100 to 1:1600. Detection of ICOS-Fc binding was performed using an anti-V5 antibody conjugated to horseradish peroxidase (HRP), followed by TMB substrate development. The enzymatic reaction was stopped after 5 min with 2N sulfuric acid, and absorbance was measured at 450 nm.

### 2.4. In Vitro Drug Release Studies

In vitro drug release studies were carried out using a multicompartment rotating cell system to evaluate PTX release kinetics from NBs decorated or not with ICOS-Fc. PTX formulations were placed in the donor compartment separated from the receiving one by a dialysis cellulose membrane (Spectra/Pore, cutoff 12,000 Da). At fixed time points, the receiving phase consisted of PBS at pH 7.4, which was removed and replaced with an equal volume of fresh PBS. PTX concentration in the withdrawn samples was determined by HPLC analysis. Results were expressed as a percentage of drug released over time and represented the mean ± standard deviation (SD) of three independent experiments.

### 2.5. Cell Lines and Culture

Experiments were carried out on JR8, M14, and D4M-3A melanoma cells. JR8 and M14 were from Dr. Pistoia (Gaslini Institute, Genoa, Italy), and D4M-3A were from SIGMA-Aldrich. D4M-3A is an adhesive metastatic melanoma murine cell line engineered to carry the BRAF^V600E^ mutation. JR8 and M14 cells were cultured in RPMI1640 medium, while D4M-3A cells were cultured in DMEM. All cultures were supplemented with 10% FBS, 100 U/mL penicillin, and 100 U/mL streptomycin and cultured in a 5% CO_2_, 37 °C incubator.

Before starting the experiment, cells were tested to be negative for mycoplasma by PCR and gel electrophoresis.

### 2.6. MTT Assay

Melanoma cells (1 × 10^3^/well) were seeded in 96-well plates for 24 h, then they were treated with free PTX, NB-PTX, ICOS-Fc-NB-PTX or empty NB using 1–100 nM as the concentration range used. MTT assay was performed to assess cell viability after 72 h of treatment. The cell proliferation reagent MTT was used, as described by the manufacturer’s protocol. Cells that had received no drug, as a control, were normalized to 100%, and the readings from treated cells were expressed as a percentage of viability inhibition. Four replicates were used to determine each data point; the results are expressed as mean ± SEM (*n* = 6).

To evaluate cell viability at the time used in the invasion test (6 h), the Crystal Violet assay was used. Cells (8 × 10^3^/well) were seeded into 96-well plates and treated for 6 h with the compounds free PTX, NB-PTX, ICOS-Fc-NB-PTX, or empty NB used at the concentration range of 1–100 nM under study [31].

### 2.7. ICOSL Analysis

Surface expression of ICOSL in D4M-3A cells was evaluated by flow cytometry (Attune NxT, Thermo-Fisher, Waltham, MA, USA) using phycoerythrin (PE)-conjugated anti-ICOSL (BioLegend, San Diego, CA, USA).

### 2.8. Migration and Invasion Assay

Migration was studied with the wound healing assay. D4M-3A cells were plated onto six-well plates (1 × 10^3^/well) and grown to confluence. Cells were left for 12 h with FBS-free medium (to prevent cell proliferation). Cell monolayers were carefully wounded by scratching with a sterile plastic pipette tip along the diameter of the well. Cells were washed twice with FBS-free medium and then incubated with culture medium in the absence or presence of the tested substances. Five fields of each of the three wounds were analyzed per condition and were photographed immediately after the scratch had been made (0 h) and 24 h later to monitor cell movement into the wounded area. The wounded area closure was calculated as (1 − (scratch width of the treated group/scratch width of the control group)) × 100%.

Invasion assays were carried out on M14 and D4M-3A melanoma cells using a 48-well Boyden chamber (1 × 10^3^/well). In the first set of experiments, cells treated with ICOS-Fc or ICOS-Fc-NB (0.05–2 µg/mL) were plated onto the apical side of 50 µg/mL Matrigel-coated filters (8.2 mm diameter and 5 µm pore size) in a medium containing 10% FBS with or without formulations under study. A medium containing FBS 20% was placed in the basolateral chamber as a chemoattractant. The chamber was incubated at 37 °C under 5% CO2. After 6 h, the cells on the apical side were wiped off with PBS. Cells on the bottom of the filter were stained with Crystal Violet and counted with an inverted microscope (magnification 20×). The results are expressed as the percent invasion inhibition vs. control of migrated cells from all the wells of each quadruplicate filter ± SEM (*n* = 5). The control invasion was 45 ± 4 and 51 ± 5 cells for M14 and D4M-3A, respectively.

In the second set of experiments, cells were treated with PTX (at the range 3–100 nM) or NB-PTX (at the range 3–100 nM) or ICOS-FC-NB-PTX (with PTX at the range 3–100 nM and ICOS-Fc at the range 0.019–5.7 µg/mL).

### 2.9. Evaluation of NB and ICOS-Fc-NB Internalization

The NB and ICOS-Fc-NB internalizations were determined in D4M-3A melanoma cells by using nanoformulations loaded with fluorescent 6-coumarin. We followed a previously published protocol [32], and the green fluorescence of 6-coumarin was examined by using fluorescence microscopy (454 nm).

### 2.10. Animals

C57BL/6 Wild-type (WT) male mice (Charles River Laboratories) were bred under pathogen-free conditions in the animal facility of the University of Piemonte Orientale, Department of Health Sciences. All experimental procedures were performed according to European Guidelines and our institution’s ethics commission. A detailed study protocol, including the research question, key design features, and analysis plan, was prepared prior to the experiment and approved by the Bioethics Committee for Animal Experimentation of UPO and Ministero della Salute, Rome (Authorization n° 241/2022-PR; Risp. a prot. DB064.76).

Experiments started at T0, injecting D4M-3A cells subcutaneously into the shaved back of 9-week-old male mice (1 × 10^6^ D4M-3A cells resuspended in 100 µL of PBS). After 7 days (T7), the tumor mass was visible and palpable, so measurement acquisitions and treatment administration were started.

A total of 28 mice were randomized into four experimental groups (7 mice per group), across two independent experimental runs, using stratified block randomization based on baseline tumor volume to ensure homogeneous distribution. Each mouse was considered the experimental unit, and no inclusion or exclusion criteria were applied during the experiments. At T7, each group separated into different cages received one of the following treatments: PBS, ICOS-Fc-NB (ICOS-Fc: 100 µg/mL), NB-PTX (200 µg/mL), or ICOS-Fc-NB-PTX. The drug preparations were administered every four days (T7, T11, T15, and T19) in a volume of 100 µL by intravenous (IV) injection into the caudal vein (dose: 1 mg/kg).

The theoretical volume of tumors was measured every two days starting from T7 with the following formula: theoretical volume = greater length × shorter length × shorter length, and represented as average ± SE.

At the endpoint (T21), mice were sacrificed by cervical dislocation, and the real volume of the tumor mass was measured, and the lungs and spleens were collected under sterile conditions. The tumor mass was weighed and measured in all its dimensions (height, length, and width) to obtain the real final volume, whereas the lungs and spleens were only weighed. For histological analyses, the lungs and spleens were placed into biocassettes and immersed in buffered formalin to be fixed and embedded in paraffin blocks; the tumor mass was instead cut and put half in biocassettes and immersed in buffered formalin and processed for paraffin embedding, and half inside cryovials for liquid nitrogen to be stored afterwards at −80 °C. The study was conducted without blinding; all investigators were aware of the group allocation at all stages (allocation, conduct of the experiment, outcome assessment, and data analysis). All experimental procedures were conducted to minimize pain and distress. Animals were handled by trained personnel and provided with environmental enrichment and monitored daily for body weight, tumor growth, behavior, and signs of pain or distress. Mild, transient local reactions at the injection site were observed as expected, with no severe or unexpected adverse events. Humane endpoints included tumor volume exceeding 1.5 cm in diameter, significant weight loss (>15–20%), or signs of severe distress, with monitoring frequency increased when animals approached these criteria.

### 2.11. Immunohistochemistry for CD31 and Ki-67

C57BL/6 formalin fixed and paraffin embedded tumor tissues were cut with a microtome (thickness 4–5 µm) and re-hydrated with decreasing concentrations of ethanol in water. The samples were then treated with 5% normal goat serum in PBS for 1 h in order to block nonspecific sites to which the primary antibody could bind. Samples were treated with citrate buffer for antigen retrieval, and endogenous peroxidases were blocked with 3% H_2_O_2_. To detect CD31 and Ki-67 expression, the primary antibodies used were a polyclonal rabbit anti-CD31 (diluted 1:50) or a monoclonal rabbit anti-Ki-67 antigen (diluted 1:200) and were incubated overnight at 4 °C in a humid chamber. Since Ki-67 is a nuclear antigen, it was necessary to permeabilize the samples by using PBS + 0.1% Triton X-100. The secondary antibody used was an anti-rabbit-HRP produced in goat (Thermo Fisher Scientific, Waltham, MA, USA), diluted 1:500 in PBS, and revealed with 3,30-diaminobenzidine (DAB). Successively, samples were counterstained with hematoxylin, dehydrated with increasing concentrations of ethanol in water, and mounted on cover slips. The sections were then acquired by a slide scanner (Axioscan, Zeiss, Oberkochen, Germany) and analyzed. Tumor microvessel density (TMD) was measured by evaluating the CD31-positive area, the numbers of positive cells for Ki-67, and the total tumor area per field upon slide scanning, as previously described [33]. The number of Ki67-positive cells was evaluated in at least ten different fields by manual counting.

### 2.12. Statistical Analysis

The sample size for the in vivo study was calculated to detect a statistically significant difference between group means, with 80% power and a 5% significance level, using an unpaired *t*-test. Statistical analysis was performed on InStat (GraphPad Prism Software, version 6.01). Comparisons among multiple groups required an ANOVA test, followed by an unpaired *t*-test for independent group values and Bonferroni adjustments. Results with *p*-value < 0.05 were considered statistically significant.

## 3. Results

### 3.1. Characterization of NB Nanoformulations

NB formulations were in vitro characterized by DLS analysis, and the physico-chemical parameters are shown in Table 1. For all the NB formulations, the average size was around 300 nm, and the surface charge was positive due to the presence of a chitosan shell. Analysis of zeta potential showed that these values were sufficiently high to avoid the aggregation of the NBs. Moreover, good values (about 0.20) for the polydispersity index, a measure of the uniformity of the size distribution of a nanoparticle population, were observed for all the samples.

Loading PTX resulted in only a slight increase in NB average diameter. The conjugation of ICOS-Fc to the chitosan shell of the NBs did not significantly modify their size. At the same time, a decrease in the zeta potential value of about 16% and 12.5% was observed for empty and PTX-loaded NBs, respectively.

**Figure 1:**
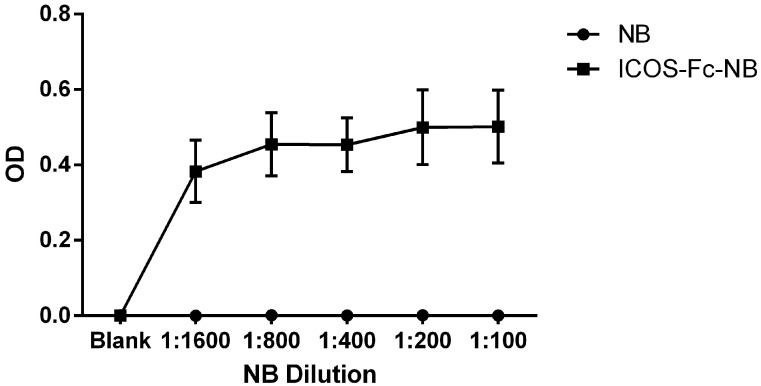
ELISA assay results obtained using ICOSL-HIS as capture protein. The ICOS-Fc-NB and the blank were assessed in different dilutions, ranging from 1:1600 to 1:100. The graph shows the mean ± SEM of three independent experiments.

The presence of ICOS-Fc on the surface of the NBs was demonstrated by ELISA assay (Figure 1). NBs decorated with ICOS-Fc showed a positive signal in terms of optical density (OD) at the end of the ELISA test in all the tested dilutions, while the blank did not show any positive signal.

**Figure 2:**
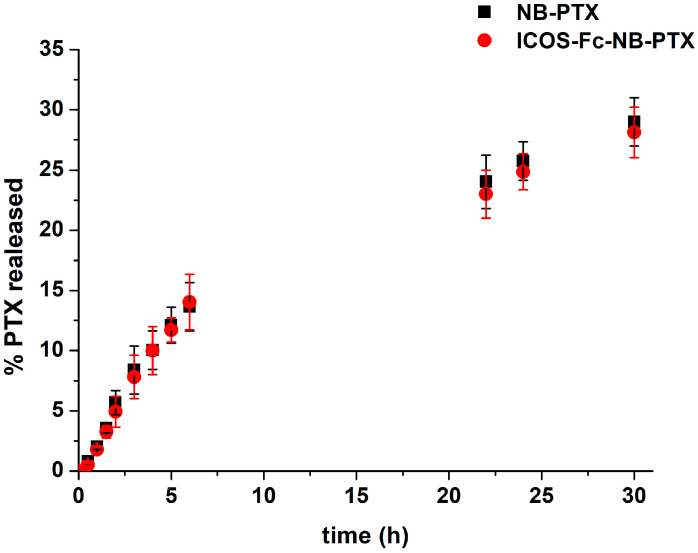
In vitro release kinetics of PTX from PTX-loaded NB conjugated or not with ICOS-Fc.

NBs were able to load PTX with high encapsulation efficiency (about 95%) and a loading capacity of 5.10%. PTX was released from the NB with slow and prolonged in vitro release kinetics over time. Figure 2 shows that, after 6 h, less than 15% of the drug was released, with no differences between NB-PTX and ICOS-Fc-NB-PTX. After 30 h, the percentage of PTX released reached about 30% for both the formulations. The absence of the initial burst effect demonstrated the PTX incorporation in the NB core. The results of the in vitro release studies best fit the Higuchi release model, indicating that the drug is released by diffusion through the polymer shell. The release profile was not influenced by the presence of ICOS-Fc conjugated to the NB surface.

A good colloidal physical stability of the NB was observed after storage of the nanoformulations at 4 °C. Indeed, no aggregation phenomena or significant modification of the physico-chemical characteristics occurred up to 6 months. Moreover, no decrease in PTX concentration in the NBs was observed over time.

### 3.2. In Vitro Cell Assays

We evaluated the effect of the different drug formulations on the viability and migration of human JR8 and M14 melanoma cells and mouse D4M-3A melanoma cells. The two human melanoma cell lines, JR8 and M14, were selected for this study based on previous results from our laboratories demonstrating that ICOS-Fc inhibits their migration [34]. The choice of a murine cell line, such as the engineered BRAF^V600E^-mutated D4M-3A, was dictated by the observation that this cell line, carrying the mutation that occurs in nearly 50% of human melanomas, can recapitulate the features of the human disease in immunocompetent animal models [35]. This characteristic is extremely important for this study, which aims to further investigate the effects of the immunomodulatory molecule ICOS-Fc. ICOSL expression was then tested in D4M-3A cells by flow cytometry, identifying two different subclones with low (1.34 mean fluorescence intensity—ratio, MFI-R) and high (2.99 MFI-R), respectively. The subsequent in vitro experiments were always performed in cells expressing ICOSL levels greater than 2MFI-R, as we had previously found this to be the cutoff level for detecting the ICOS-Fc effect in vitro.

#### 3.2.1. Viability

**Figure 3:**
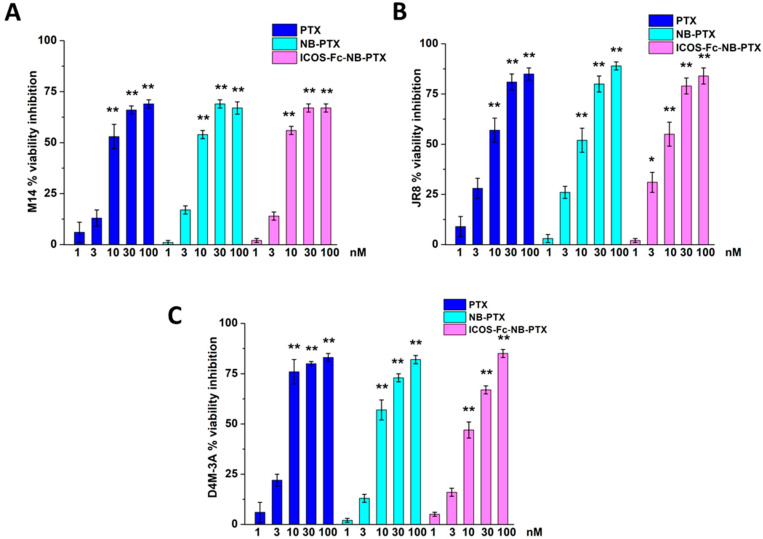
Viability (MTT assay) in M14 (**A**), JR8 (**B**), and D4M-3A (**C**) exposed to PTX, NB-PTX, and ICOS-Fc-NB -PTX at the indicated concentrations after 72 h of treatment. Results are expressed as a percentage of viability inhibition vs. control. The data are mean ± SEM (*n* = 6). * *p* < 0.05 and ** *p* < 0.01 vs. untreated cells.

The three melanoma cell lines were treated or not with free PTX, free ICOS-Fc, empty NB, NB-PTX, ICOS-Fc-NB, or ICOS-Fc-NB-PTX at the concentration range between 1 nM and 100 nM, and cell viability was assessed after 72 h by MTT. Results showed that, in all cell lines, inhibition of cell viability was similar at each titration point using either PTX or NB-PTX or ICOS-Fc-NB-PTX (Figure 3). By contrast, no effect on cell viability was detected using either free ICOS-Fc, empty NB, or ICOS-Fc-NB.

#### 3.2.2. Cell Migration

The effect of each drug formulation on cell migration was evaluated using the Boyden chamber assay. Since the two human cell lines showed similar results in viability inhibition, we chose M14 for further investigations on migration, next to the murine D4M-3A also used for in vivo studies. In preliminary experiments, we assessed the effectiveness of ICOS-Fc decorating the NB surface by comparing the ability of free ICOS-Fc and ICOS-Fc-NB to inhibit the migration of M14 and DM4-3A cells, at concentrations ranging from 0.05 to 2 µg/mL in a Boyden chamber assay.

**Figure 4:**
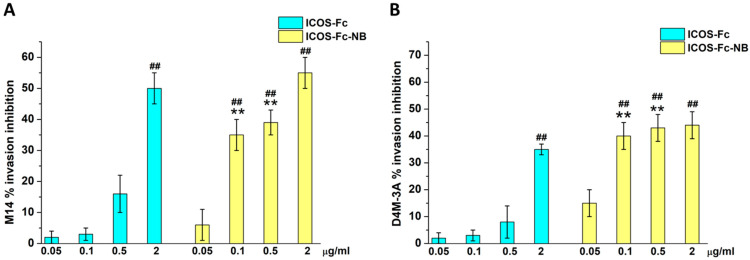
Invasion assay in M14 (**A**) and D4M-3A (**B**) exposed to ICOS-Fc or ICOS-Fc-NB at the indicated concentrations. Cells were treated for 6 h in a Boyden chamber. Results are expressed as a percentage of invasion inhibition compared to the control. The data are mean ± SEM (*n* = 6). ## *p* < 0.01 ICOS-Fc or ICOS-Fc-NB vs. control; ** *p* < 0.01 ICOS-Fc-NB vs. ICOS-Fc.

Results showed that free ICOS-Fc was able to inhibit cell invasion of both cell lines only at the highest concentration, whereas ICOS-Fc-NB was already effective at 0.1 µg/mL (Figure 4).

**Figure 5:**
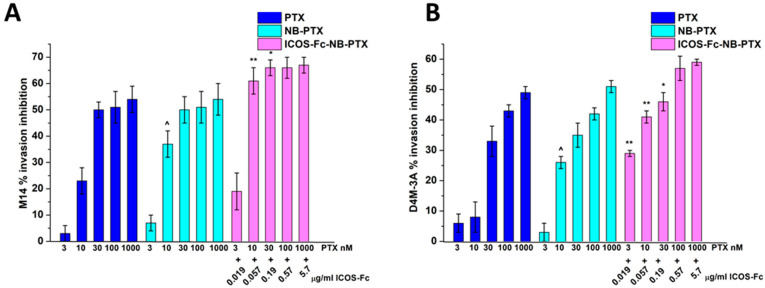
Invasion assay in M14 (**A**) and D4M-3A (**B**) exposed to PTX or NB-PTX or ICOS-Fc-NB- PTX at the indicated concentrations. Cells were treated for 6 h in a Boyden chamber. Results are expressed as a percent of invasion inhibition vs. control. The data are mean ± SEM (*n* = 6). ^ *p* < 0.05 NB-PTX vs. PTX; ** *p* < 0.01 ICOS-Fc-NB-PTX vs. NB-PTX and PTX; * *p* < 0.05 ICOS-Fc-NB-PTX vs. NB-PTX and PTX.

Then, we compared the migration of M14 and D4M-3A cells treated or not with titrated amounts of free PTX, NB-PTX, or ICOS-Fc-NB-PTX. Results in Figure 5 showed that all drug formulations inhibited cell invasion, and ICOS-Fc-NB-PTX exhibited higher inhibition than PTX and NB-PTX at most concentrations. In contrast, NB-PTX induced higher inhibition than PTX only at 10 nM.

**Figure 6:**
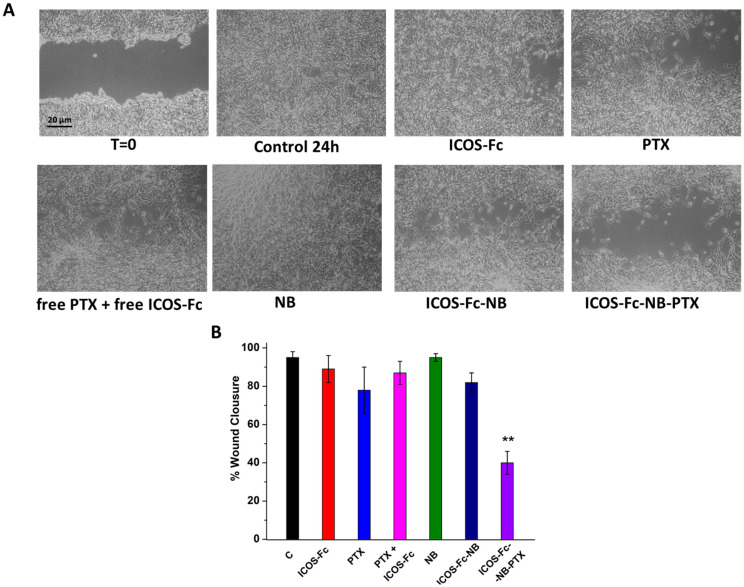
Wound healing assay in D4M-3A cells untreated or exposed to 0.057 µg/mL free ICOS-Fc, 10 nM free PTX, 10 nM free PTX plus 0.057 µg/mL free ICOS-Fc, empty NB, ICOS-Fc-NB (ICOS-Fc 0.057 µg/mL final concentration), and ICOS-Fc-NB-PTX (ICOS-Fc 0.057 µg/mL and PTX 10 nM final concentrations). (**A**) Microphotographs of the wounded area were taken immediately after the scratch (0 h) and after 24 h, in order to monitor cell migration into the wounded area. (**B**) The graph shows mean ± SD (*n* = 6) of assay endpoints measured by calculating the reduction in the width of the wound after 24 h and compared to T0, which is set at 100%. Results are expressed as a percent of invasion inhibition vs. control. The data are mean ± SEM (*n* = 6). ** *p* < 0.01 ICOS-Fc-NB-PTX vs. all treatments.

To confirm these results, we compared cell migration using the wound healing assay in D4M-3A cells treated or not with 0.057 µg/mL free ICOS-Fc, 10 nM free PTX, 10 nM free PTX plus 0.057 µg/mL free ICOS-Fc, empty NB, ICOS-Fc-NB (ICOS-Fc 0.057 µg/mL final concentration), and ICOS-Fc-NB-PTX (ICOS-Fc 0.057 µg/mL and PTX 10 nM final concentrations). Results (Figure 6) demonstrated that only the treatment with ICOS-Fc-NB-PTX significantly inhibited cell migration compared to all the other treatments.

#### 3.2.3. NB and ICOS-Fc-NB Internalization

**Figure 7:**
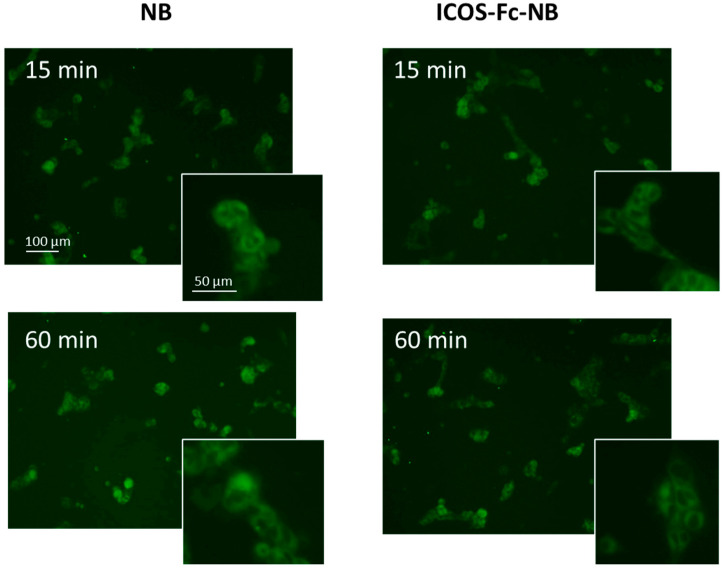
Internalization of 6-coumarin-NB and 6-coumarin-ICOS-Fc-NB in D4M-3A cells at 15 and 60 min. Green fluorescence of 6-coumarin was examined by using fluorescence microscopy (454 nm).

The cellular uptake of NBs and ICOS-Fc-NB were evaluated in D4M-3A cells at 37 °C by fluorescence microscopy using fluorescent-labeled 6-coumarin nanoformulations. As shown in Figure 7, both NBs and ICOS-Fc-NB were internalized within 15 min into both cell lines and showed similar values at 60 min.

### 3.3. In Vivo Assays

To assess the anticancer effect of the different drug formulations in vivo, we used 9-week-old C57BL/6 male mice injected subcutaneously with 1 × 10^6^ DM4-3A cells. Since ICOSL expression tends to decrease in DM4-3A cells upon prolonged culture, we chose to use DM4-3A cells expressing low levels of ICOSL in order to minimize the variability in the results due to changes in the ICOSL expression level.

After 7 days from cell injection (T7), when tumors were palpable, mice were injected IV every 4 days with PBS, NB-PTX, ICOS-Fc-NB, or ICOS-Fc-NB-PTX, and tumor growth was monitored every other day using a caliper. Mice were sacrificed after 14 days of treatment, i.e., 21 days after cell injection (T21).

**Figure 8:**
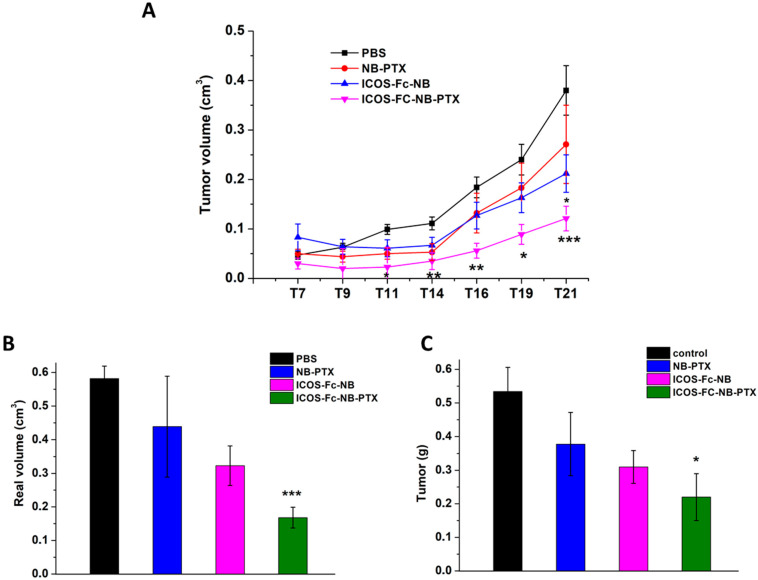
Effects of the treatments on tumor growth time progression in vivo and on final weight and volume ex vivo. Data are expressed as mean ± SEM (*n* = 7). (**A**) Kinetics of the theoretical tumor volume state of mice treated with PBS, NB-PTX, ICOS-Fc-NB, and ICOS-Fc-NB-PTX. Statistical analysis: ICOS-Fc-NB-PTX vs. PBS: * *p* < 0.05; ** *p* < 0.005; *** *p* < 0.0005. ICOS-Fc-NB vs. PBS. (**B**) Final real tumor volume measured ex vivo. ICOS-Fc-NB-PTX vs. PBS: *** *p* < 0.0005. (**C**) Final tumor weight measured ex vivo. ICOS-Fc-NB-PTX vs. PBS: * *p* < 0.05.

In vivo monitoring of tumor growth showed that the estimated tumor volumes were significantly lower in mice treated with ICOS-Fc-NB-PTX compared to control mice from T11, with the best result achieved at T19 (effect size (d) = 2.19; confidence interval (CI) = 0.82–3.55). By contrast, treatment with ICOS-Fc-NB significantly decreased the tumor volume compared to control mice only at T21, and NB-PTX had no significant effect (Figure 8A). Analysis of tumors excised at the T21 endpoint showed that the volume (d = 4.6; CI = 2.64–6.56) and weight (d = 1.67; CI = 0.43–2.91) of the tumors were significantly lower in the mice treated with ICOS-Fc-NB-PTX than in control mice. In contrast, differences from controls were not significant in mice treated with NB-PTX or ICOS-Fc-NB (Figure 8B,C).

**Figure 9:**
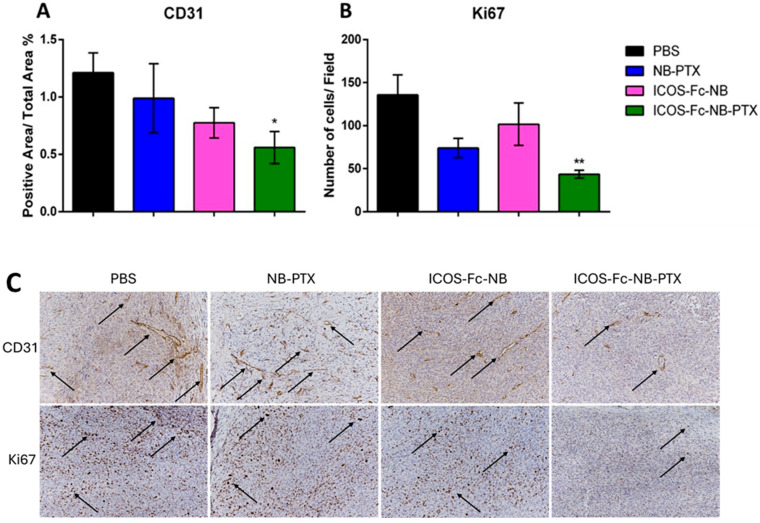
Effects of the treatments on proliferation and angiogenesis markers. Data are expressed as mean ± SEM (*n* = 7). (**A**) Single cell positivity for Ki67 tested by IHC. (**B**) The ratio of CD31-positive area over total area percentage, tested by IHC. (**C**) Representative images of IHC staining for CD31 and Ki67, arrows indicate the positive signal. Statistical analysis: ICOS-Fc-NB-PTX vs. PBS * *p*-value < 0.05; ** *p* < 0.005.

Immunohistochemical analyses were performed on the excised tumors to assess the number of Ki67^+^ tumor cells, which mark proliferating cells, and CD31^+^ blood vessels. Results showed that Ki67^+^ tumor cells (d = 2.08; CI = 0.73–3.43) and CD31^+^ blood vessels (d = 1.56; CI = 0.34–2.78) were significantly lower in the tumors excised from the mice treated with ICOS-Fc-NB-PTX than in those excised from the control mice, whereas no significant effects were detected in mice treated with NB-PTX or ICOS-Fc-NB (Figure 9).

**Figure 10:**
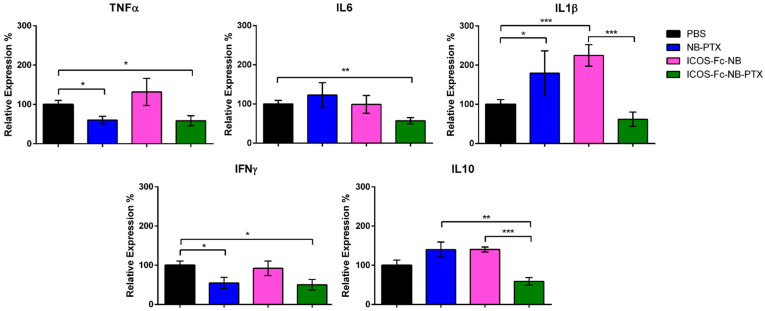
Effects of the treatments on the expression of cytokine mRNA. Relative expression of TNFα, IL6, IL1β, IFNγ, and IL10 was assessed at the mRNA level by RT PCR in lysates obtained from the tumor masses. The data are mean ± SEM (*n* = 7). * *p*-value < 0.05; ** *p* < 0.01; *** *p* < 0.005.

The expression of several cytokines was assessed at the mRNA level by RT PCR in lysates obtained from frozen tumor masses. Results showed that, compared to the control, treatment with ICOS-Fc-NB-PTX significantly decreased the expression of TNFα (d = 1.35; CI = 0.17–2.53), IL-6 (d = 1.72; CI = 0.47–2.97), and IFNγ (d = 3.04; CI = 1.43–4.65). In contrast, NB-PTX decreased TNFα (d = 1.52; CI = 1.05–3.95) and IFNγ (d = 2.5; CI = 0.29, 2.75) but increased IL-1β (d = 1.24; CI = 0.08–2.40), while ICOS-Fc-NB increased IL-1β (d = 2.22; CI = 0.83–3.61). Moreover, tumors treated with ICOS-Fc-NB-PTX exhibited significantly lower levels of IL-10 (d = 1.34; CI = 0.18–2.50) compared to those treated with ICOS-Fc-NB or NB-PTX (Figure 10).

**Figure 11:**
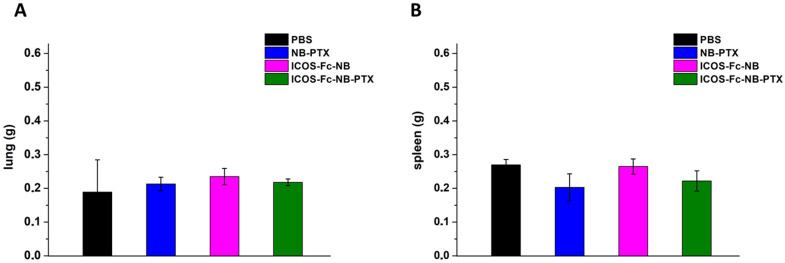
Effects of the treatments on the animal weight of lungs (**A**) and spleen (**B**). The data are mean ± SEM.

All treatments were well tolerated by the animals, and no animals were excluded from calculations, as no significant weight loss of the total body, lungs, or spleen was detected in any animal group (Figure 11).

## 4. Discussion

Currently, PTX is not the first-line treatment against melanoma in clinical practice [36]. However, its anticancer activity related to immunogenic cell death might be boosted by immunomodulatory agents. Specifically, the combination of conventional chemotherapies with immunotherapies could improve the therapeutic outcome of melanoma [37]. This work shows that a combined therapy using PTX loaded into chitosan NBs decorated with ICOS-Fc displays a strong anti-neoplastic activity in vivo against a mouse melanoma model using the BRAF^V600E^-mutated murine D4M-3A cell line.

The choice of D4M-3A cells for our in vitro and, above all, in vivo experiments lies in their ability to reproduce the human disease in an immunocompetent experimental environment. The BRAF^V600E^ mutation is present in nearly 50% of all human melanomas, leading to a constitutive activation of MAPK signaling [38]. While human melanoma cell lines carrying the BRAF^V600E^ mutation are well characterized, the frequent use of xenografts in immunodeficient mice restricts the ability to study tumor–host immune interactions in a fully immunocompetent system. The D4M-3A cell line, derived from a conditional mouse model of metastatic melanoma [35], carries this mutation, which is typical of humans, thus closely mirroring the human disease. Indeed, the D4M-3A cell line in vitro recapitulates the human BRAF^V600E^ melanoma cells in vivo, for instance, by expressing the same markers found in vivo [35]. The presence of the BRAFV600E mutation enables studies with BRAF inhibitors (BRAFi) and elucidates the molecular mechanisms underlying BRAFi resistance [39]. Moreover, for our purpose, D4M-3A cells can represent a good model to study the effect of ICOS-Fc- based nanoformulations, since we demonstrate the expression of ICOSL on their surface. Furthermore, since D4M-3A cells can be transplanted into syngeneic mice (i.e., C57BL/6 Wild-type), this also allows immunological studies in immunocompetent animal models. For instance, it is possible to study the role of immune cells during BRAFi therapies [40] or explore the effects of the association between target therapy and immunotherapy [41]. An intact immune system is essential for our present study, which aims to investigate the effects of the immunomodulatory molecule ICOS-Fc. Overall, the D4M-3A cell line serves as a clinically relevant model for metastatic melanoma across both in vitro and in vivo experimental settings.

Previously, we demonstrated that the loading of a single drug in a nanosized delivery system can enhance the anti-tumor effect. In this context, either PTX loaded in cyclodextrin-based nanosponges [9] or ICOS-Fc loaded in polymer nanoparticles (PLGA or cyclodextrin-based nanoparticles) [18] are highly effective in vivo against the non-BRAF mutated B16-F10 melanoma, which is less aggressive than the D4M-3A model used in the present work. Moreover, combined three-drug therapy using nanoemulsions loaded with ICOS-Fc plus sorafenib and temozolomide showed increased anti-tumor activity in vivo against the B16-F10 melanoma [22]. In all these settings, loading of the drugs into the nanoparticles substantially increased their anti-neoplastic activity, and this was particularly crucial for ICOS-Fc, which does not show any anti-tumor activity in vivo when used as a free molecule.

The anti-neoplastic activity of ICOS-Fc loaded into nanoparticles against established B16 melanoma has been ascribed to multifaceted effects, but a pivotal role is ascribed to inhibition of tumor angiogenesis, which has been detected in all experimental settings. Inhibitory effects may accompany this effect on the immunosuppressive tumor microenvironment, which partly depends on the nanoparticle type used to treat the mice. Finally, ICOS-Fc can have direct effect on tumor cells expressing ICOSL by inhibiting tumor cell proliferation, migration, and metastatization which has been detected both in vitro and in vivo. Moreover, beside triggering ICOSL, ICOS-Fc also interferes with the binding between endogenous ICOS and ICOSL, working as a soluble decoy receptor that prevents ICOS-mediated costimulatory signaling in T cells. This blockade limits the activation and maintenance of ICOS^+^ regulatory T cells (Tregs), which generally contribute to the immunosuppressive environment within tumors. As a result, ICOS-Fc treatment can shift the immune balance toward a more active anti-tumor response, enhancing effector T-cell function and reducing tumor immune evasion. Interestingly, it is well known that certain chemotherapeutic drugs, such as PTX, can induce the immunogenic cell death (ICD), a specialized form of apoptosis that enables immune-competent hosts to trigger a specific immune response against tumor cells [42,43]. Thus, considering our experimental results, we can suggest that PTX may initiate an immunogenic remodeling of the tumor microenvironment, while ICOS-Fc further amplifies this response.

The present work used D4M-3A cells expressing low levels of ICOSL for in vivo experiments since these cells tend to lose ICOSL expression over time, which might be a confounding factor. Thus, these experiments highlight the effects of ICOS-Fc on the tumor microenvironment and underestimate the direct effects on tumor cells, which is nevertheless valuable since it releases the tumors potentially targeted by ICOS-Fc from the need to express ICOSL.

Chitosan-shelled NBs are demonstrated to be a suitable platform for the co-delivery of PTX and ICOS-Fc thanks to the versatility of this nanostructure, which easily allows for the incorporation of lipophilic molecules in the inner core and the conjugation of ligands to the polymer shell. In addition, previous studies have shown that NBs are a promising nanocarrier to enhance the anticancer effect of chemotherapeutics, exploiting the EPR effect. The high deformability and flexibility of this type of nanovesicles would allow the extravasation of relatively large nanosystems (about 300 nm) through the irregular microvessels of the tumor, favoring their entrapment into the tumor mass, which increases the local effect of the drugs and lowers their systemic adverse effects [44,45]. However, the NB sizes could be optimized by modifying the nanostructure’s interfacial and shell components and by tuning the preparation protocol [46]. Other authors reported the encapsulation of PTX in multifunctional NBs for drug delivery and imaging of prostate cancer [47,48,49] and in targeted NBs for selective lung cancer treatment [50,51] or breast cancer [52]. A crucial and new finding of the present study is that the anti-neoplastic activity of ICOS-Fc is maintained when ICOS-Fc is linked to chitosan on the NB surface. The good anti-tumor activity of ICOS-Fc conjugated on the NB surface might be ascribed to its high valence compared to the Ig-like bivalency of ICOS-Fc, which is expected to increase the level of ICOSL crosslinking and stimulation. In line, ICOS-Fc-NB displayed a higher ability to inhibit tumor cell migration in vitro than free ICOS-Fc.

The in vivo experiments showed that ICOS-Fc present on the surface of ICOS-Fc-NB exerts a mild anti-neoplastic activity since inhibition of tumor growth was significant only at the last time point, whereas the efficacy of NB-PTX was marginal due to the low dose in this experimental setting. The ultra-low dose of PTX (1 mg/kg) administered in the in vivo experiments was selected to enable the understanding of the anticancer role of the co-delivered ICOS-Fc and their synergistic therapeutic efficacy. Moreover, it was shown that PTX at ultra-low doses may exert immunomodulatory effects, improving the clinical outcome of metastatic melanoma [53]. By contrast, the therapeutic effect was substantially increased using the ICOS-Fc-NB-PTX combination, which indicates that the two drugs have additive anti-neoplastic effects when co-delivered in the same nanoparticles. The different efficacy of the NB formulations is not ascribable to differences in the physico-chemical parameters between them since they displayed similar sizes, polydispersity index, and zeta potential. Moreover, ICOS-Fc-NB-PTX exposed the same amount of ICOS-Fc as ICOS-Fc-NB, and the release of PTX from the two NB formulations, decorated or not, was similar.

Similar additive effects were previously obtained using Intralipid© nanoemulsions loaded with ICOS-Fc together with the tyrosine kinase inhibitor sorafenib and the anti-angiogenic agent temozolomide [22]. These nanoemulsions carried suboptimal amounts of the three drugs because of their limited loading capacity, so that significant anti-tumor activity was detected only in mice treated with the combination of all three drugs.

The additive effect of PTX and ICOS-Fc may be ascribed to the anti-angiogenic effect displayed by both drugs, as shown by immunohistochemistry assessing CD31^+^ blood vessels. This anti-angiogenic effect might also contribute to the decreased proliferation rate of the tumor cells, detected as Ki67 positivity in the sections obtained from the mice treated with ICOS-Fc-NB-PTX.

However, direct effects on the tumor cells cannot be ruled out since PTX is known to exert a strong anti-proliferative activity due to its capacity to inhibit microtubule dynamics and to perturb mitosis [54], and our in vitro experiments show that cytotoxicity is increased in NB-PTX compared to free PTX. By contrast, these experiments did not detect direct anti-proliferative activity using ICOS-Fc-NB, and cytotoxicity was similar in ICOS-Fc-NB-PTX and NB-PTX. This marks a difference with our previous experiments using cyclodextrin nanoparticles loaded with ICOS-Fc, which showed a direct cytotoxic effect in vitro. The difference might be ascribed to the different amounts of ICOS-Fc carried by the two types of nanoparticles and by the loading strategies exploited. Indeed, ICOS-Fc was loaded in the cyclodextrin nanosponge matrix at 1 mg/mL concentration while NB were chemically conjugated with ICOS-Fc (100 μg/mL). In addition, a different susceptibility to the ICOS-Fc mediated cytotoxicity of B16-F10 cells used in the previous experiments [21], and D4M-3A cells used in this work could be observed.

Finally, ICOS-Fc and PTX may also cooperate in modulating the immune components of the tumor microenvironment since treatment with the different drug formulations induced different changes in the cytokine expression pattern in the tumor mass. In particular, a comparison of tumors treated with ICOS-Fc-NB-PTX or NB-PTX showed that the former express low levels of IL-10 and IL-6, whereas the latter high levels of IL-1β, and both low levels of TNFα and IFNγ. The low levels of TNFα and IFNγ induced by both treatments might be ascribed to tumor debulking. The high level of IL-1β induced by NB-PTX might be due to debulking in the absence of ICOS-Fc, which can modulate IL-1β production in the tumor microenvironment. The low levels of IL-10 and IL-6 induced by ICOS-Fc-NB-PTX might be ascribed to inhibition of ICOS triggering in T cells, which is known to induce production of these cytokines, and may have an impact on decreasing the immunosuppression and the angiogenesis mediated by these cytokines, respectively. This result is in line with that previously obtained with ICOS-Fc loaded into cyclodextrin nanoparticles, which decreased IL-10 expression in B16-F10 melanoma tumors. Intriguingly, this effect was partly independent of T cells since it was also detected in ICOS-deficient mice, suggesting a key role of inflammatory cells. Therefore, treatment with ICOS-Fc-NB-PTX might benefit from the association with standard checkpoint inhibitors capable of potentiating the effector T cell response.

In conclusion, the potential of chitosan NB to be exploited as a system for co-delivery of immunomodulatory molecules in support of chemotherapeutics was highlighted. The combined treatment with PTX loaded into chitosan NBs decorated with ICOS-Fc displays a substantial anti-melanoma effect in vivo against a highly aggressive BRAF-mutated tumor. These are preliminary data and deserve further studies aimed at optimizing doses and posology, and to assess the possible combination of ICOS-Fc-NB-PTX with standard checkpoint inhibitors. The findings and their implications should be discussed in the broadest context possible. Future research directions may also be highlighted.

## Figures and Tables

**Table 1:** Physico-chemical characteristics of NB formulations. The results are reported as the average of three measurements carried out after independent experiments.

	Average Diameters ± SD (nm)	Polydispersity Index ± SD	Zeta Potential ±SD (mV)
Blank NB	305.3 ± 10.6	0.20 ± 0.02	32.14 ± 1.40
NB-PTX	310.7 ± 11.8	0.22 ± 0.03	30.90 ± 1.25
ICOS-Fc-NB	304.5 ± 8.5	0.21 ± 0.02	26.86 ± 1.58
ICOS-Fc-NB-PTX	308.2 ± 9.4	0.22 ± 0.02	27.05 ± 1.06
Fluorescent NB	306.8 ± 12.2	0.22 ± 0.01	32.05 ± 1.63
Fluorescent ICOS-Fc-NB	305.99 ± 12.16	0.24 ± 0.03	26.93 ± 1.36

## Data Availability

The original contributions presented in this study are included in the article. Further inquiries can be directed to the corresponding authors.

## Abbreviations

The following abbreviations are used in this manuscript:

BRAF	B-Raf proto-oncogene, serine/threonine kinase
BRAF^V600E^	BRAF mutated gene in which valine (V) is substituted by glutamic acid (E) at amino acid 600
CCl_4_	Carbon tetrachloride
CD31^+^	Cluster of differentiation 31
CDNS	β-cyclodextrin nanosponges
CI	Confidence Interval
CTLA-4	Cytotoxic T-lymphocyte antigen 4 r
d	Effect size
DAB	3,30-diaminobenzidine
DLS	Dynamic Light Scattering
DMEM	Dulbecco’s Modified Eagle Medium
DMSO	Dimethyl sulfoxide
EDC	3-dimethylaminopropyl)carbodiimide
ELISA.	Enzyme-Linked Immunosorbent Assay
EPR	Enhanced Permeability and Retention
FBS	Fetal Bovine Serum
Fc	Fragment constant portion of immunoglobulin
FDA	Food and Drug Administration
HPLC	High-Performance Liquid Chromatography
HRP	Horseradish Peroxidase
ICOS	Inducible Co-Stimulator
ICOS-Fc	recombinant protein ICOS-Fc, which combines the extracellular portion of ICOS with the fragment constant (Fc) portion of Immunoglobulin G1 (IgG1).
ICOSL	ICOS ligand
IFNγ	Interferon gamma
IgG1	Immunoglobulin G1
IHC	immunohistochemistry
IL-10	Interleukin-10
IL-1β	Interleukin-1 beta
IL-23	Interleukin-23
IL-6	Interleukin-6
IV	Intravenous
MAPK	Mitogen-Activated Protein Kinase
MTT	3-(4,5-dimethyl thiazol-2-yl)-2,5-diphenyltetrazolium bromide
NB/NBs	Nanobubble, Nanobubbles
NHS	N-Hydroxysuccinimide
OD	optical density
PBS	Phosphate-buffered saline
PD-1	Programmed cell death protein 1
PDL-1	PD-1 ligand
PLGA	poly(lactic-*co*-glycolic) acid
PTX	Paclitaxel
SEM	Standard Error of the Mean
Th17	Lymphocyte T helper, type 17
TMD	Tumor microvessel density
TNFα	Tumor necrosis factor alfa
Treg	Lymphocyte T regulatory cells
WT	Wild-type

## References

[1] Long G.V., Swetter S.M., Menzies A.M., Gershenwald J.E., Scolyer R.A. (2023). Cutaneous melanoma. Lancet.

[2] Raimondi S., Suppa M., Gandini S. (2020). Melanoma Epidemiology and Sun Exposure. Acta Derm. Venereol..

[3] Belzer A., Parker E.R. (2023). Climate Change, Skin Health, and Dermatologic Disease: A Guide for the Dermatologist. Am. J. Clin. Dermatol..

[4] Battaglia L., Scomparin A., Dianzani C., Milla P., Muntoni E., Arpicco S., Cavalli R. (2021). Nanotechnology Addressing Cutaneous Melanoma: The Italian Landscape. Pharmaceutics.

[5] Hendrix M.J.C., Seftor E.A., Margaryan N.V., Seftor R.E.B., Ward W.H., Farma J.M. (2017). Heterogeneity and Plasticity of Melanoma: Challenges of Current Therapies. Cutaneous Melanoma: Etiology and Therapy.

[6] Rausch M.P., Hastings K.T., Ward W.H., Farma J.M. (2017). Immune Checkpoint Inhibitors in the Treatment of Melanoma: From Basic Science to Clinical Application. Cutaneous Melanoma: Etiology and Therapy.

[7] Vicari A.P., Luu R., Zhang N., Patel S., Makinen S.R., Hanson D.C., Weeratna R.D., Krieg A.M. (2009). Paclitaxel reduces regulatory T cell numbers and inhibitory function and enhances the anti-tumor effects of the TLR9 agonist PF-3512676 in the mouse. Cancer Immunol. Immunother..

[8] Seyam S., Alallam B., Harif Fadzilah N., Abd Kadir E. (2025). Advances in paclitaxel nanoformulations: A systematic review of in vivo therapeutic efficacy and safety enhancements. J. Control Release.

[9] Clemente N., Argenziano M., Gigliotti C.L., Ferrara B., Boggio E., Chiocchetti A., Caldera F., Trotta F., Benetti E., Annaratone L. (2019). Paclitaxel-Loaded Nanosponges Inhibit Growth and Angiogenesis in Melanoma Cell Models. Front. Pharmacol..

[10] Huang Y., Wang K., Yu M., Zhou Q., Wang J., Chen S., Gong J., Yang M., Huang J., Zhao Y. (2025). Co-delivery paclitaxel and IR783 as nanoparticles for potentiated chemo-photothermal-immunotherapy of triple-negative breast cancer. Mater. Today Bio.

[11] Lee J., Kim J.M., Baek Y.J., Kang H., Choi M.K., Song I.S. (2025). Anticancer Activity of Paclitaxel-Loaded Mesoporous Silica Nanoparticles in B16F10 Melanoma-Bearing Mice. Pharmaceutics.

[12] Chen L., Hu M., Yan H.M., Ding X.Q., Yang Q.X., Wang L., Pan H. (2025). Dual-targeted albumin nanoparticles for the Co-delivery of low-dose paclitaxel and PCSK9 inhibitor in melanoma treatment. Mater. Today Bio.

[13] Solinas C., Gu-Trantien C., Willard-Gallo K. (2020). The rationale behind targeting the ICOS-ICOS ligand costimulatory pathway in cancer immunotherapy. ESMO Open.

[14] Amatore F., Gorvel L., Olive D. (2020). Role of Inducible Co-Stimulator (ICOS) in cancer immunotherapy. Expert Opin. Biol. Ther..

[15] Bauquet A.T., Jin H., Paterson A.M., Mitsdoerffer M., Ho I.C., Sharpe A.H., Kuchroo V.K. (2008). The costimulatory molecule ICOS regulates the expression of c-Maf and IL-21 in the development of follicular T helper cells and TH-17 cells. Nat. Immunol..

[16] Raineri D., Cappellano G., Vilardo B., Maione F., Clemente N., Canciani E., Boggio E., Gigliotti C.L., Monge C., Dianzani C. (2021). Inducible T-Cell Costimulator Ligand Plays a Dual Role in Melanoma Metastasis upon Binding to Osteopontin or Inducible T-Cell Costimulator. Biomedicines.

[17] Dianzani C., Minelli R., Gigliotti C.L., Occhipinti S., Giovarelli M., Conti L., Boggio E., Shivakumar Y., Baldanzi G., Malacarne V. (2014). B7h triggering inhibits the migration of tumor cell lines. J. Immunol..

[18] Acharya S., Sahoo S.K. (2011). PLGA nanoparticles containing various anticancer agents and tumour delivery by EPR effect. Adv. Drug Deliv. Rev..

[19] Maeda H., Fang J., Inutsuka T., Kitamoto Y. (2003). Vascular permeability enhancement in solid tumor: Various factors, mechanisms involved and its implications. Int. Immunoph..

[20] El-Kenawy A.E.M., Constantin C., Hassan S.M.A., Mostafa A.M., Neves A.F., de Araújo T.G., Neagu M., Ward W.H., Farma J.M. (2017). Nanomedicine in Melanoma: Current Trends and Future Perspectives. Cutaneous Melanoma: Etiology and Therapy.

[21] Clemente N., Boggio E., Gigliotti L.C., Raineri D., Ferrara B., Miglio G., Argenziano M., Chiocchetti A., Cappellano G., Trotta F. (2020). Immunotherapy of experimental melanoma with ICOS-Fc loaded in biocompatible and biodegradable nanoparticles. J. Control Release.

[22] Monge C., Stoppa I., Ferraris C., Bozza A., Battaglia L., Cangemi L., Miglio G., Pizzimenti S., Clemene N., Gigliotti C.L. (2022). Parenteral Nanoemulsions Loaded with Combined Immuno- and Chemo-Therapy for Melanoma Treatment. Nanomaterials.

[23] Shah R., Phatak N., Choudhary A., Gadewar S., Ajazuddin A., Bhattacharya S. (2024). Exploring the Theranostic Applications and Prospects of Nanobubbles. Curr. Pharm. Biotechnol..

[24] Baharlouei P., Rahman A. (2022). Chitin and Chitosan: Prospective Biomedical Applications in Drug Delivery, Cancer Treatment, and Wound Healing. Mar. Drugs.

[25] Zhou X., Guo L., Shi D., Duan S., Li J. (2019). Biocompatible Chitosan Nanobubbles for Ultrasound-Mediated Targeted Delivery of Doxorubicin. Nanoscale Res. Lett..

[26] Argenziano M., Spagnolo R., Cavalli R. (2025). What are the future applications of chitosan nanobubbles in drug delivery?. Expert Opin. Drug Deliv..

[27] Marano F., Rinella L., Argenziano M., Cavalli R., Sassi F., D’Amelio P., Battaglia A., Gontero P., Bosco O., Peluso R. (2016). Targeting Taxanes to Castration-Resistant Prostate Cancer Cells by Nanobubbles and Extracorporeal Shock Waves. PLoS ONE.

[28] Mossenta M., Argenziano M., Capolla S., Busato D., Durigutto P., Mangogna A., Polano M., Sblattero D., Cavalli R., Macor P. (2025). Idarubicin-loaded chitosan nanobubbles to improve survival and decrease drug side effects in hepatocellular carcinoma. Nanomedicine.

[29] Di Cintio F., Argenziano M., Scomparin A., Capolla S., Busato D., Steffè A., Mangogna A., Sblattero D., Cavalli R., Macor P. (2025). The anti-glypican 1 AT101 antibody as targeting agent to effectively deliver chitosan nanobubbles to glioblastoma cells. Nanomedicine.

[30] Fiorilli S., Pagani M., Boggio E., Gigliotti C.L., Dianzani C., Gauthier R., Pontremoli C., Montalbano G., Dianzani U., Vitale-Brovarone C. (2021). Sr-Containing Mesoporous Bioactive Glasses Bio-Functionalized with Recombinant ICOS-Fc: An In Vitro Study. Nanomaterials.

[31] Dianzani C., Monge C., Miglio G., Serpe L., Martina K., Cangemi L., Ferraris C., Mioletti S., Osella S., Gigliotti C.L. (2020). Nanoemulsions as Delivery Systems for Poly-Chemotherapy Aiming at Melanoma Treatment. Cancers.

[32] Argenziano M., Monge C., Scomparin A., Trotta F., Boscaro V., Stoppa I., Dianzani U., Pizzimenti S., Cavalli R., Dianzani C. (2025). Gemcitabine-loaded ICOS-Fc decorated nanosponges: A new chemo immunotherapy combination against pancreatic cancer. Int. J. Pharm..

[33] Gigliotti C.L., Minelli R., Cavalli R., Occhipinti S., Barrera G., Pizzimenti S., Cappellano G., Boggio E., Conti L., Fantozzi R. (2016). In Vitro and In Vivo Therapeutic Evaluation of Camptothecin-Encapsulated β-Cyclodextrin Nanosponges in Prostate Cancer. J. Biomed. Nanotechnol..

[34] Raineri D., Dianzani C., Cappellano G., Maione F., Baldanzi G.I., Clemente N., Baldone G., Boggio E., Gigliotti C.L., Boldorini R. (2020). Osteopontin, binds ICOSL promoting tumor metastasis. Commun. Biol..

[35] Jenkins M.H., Steinberg S.M., Alexander M.P., Fisher J.L., Ernstoff M.S., Turk M.J., Mullins D.W., Brinckerhoff C.E. (2014). Multiple murine BRaf(V600E) melanoma cell lines with sensitivity to PLX4032. Pigment. Cell Melanoma Res..

[36] Pham J.P., Joshua A.M., da Silva I.P., Dummer R., Goldinger S.M. (2023). Chemotherapy in Cutaneous Melanoma: Is There Still a Role?. Curr. Oncol. Rep..

[37] Yang J., Wang X., Meng Y., Zhu M., Kong F. (2025). Combination Immunotherapy for Mucosal Melanoma: Molecular Mechanism, Research Status, and Future Directions. Curr. Treat. Options Oncol..

[38] Amaral T., Sinnberg T., Meier F., Krepler C., Levesque M., Niessner H., Garbe C. (2017). The mitogen-activated protein kinase pathway in melanoma part I—Activation and primary resistance mechanisms to BRAF inhibition. Eur. J. Cancer..

[39] Cucci M.A., Grattarola M., Monge C., Roetto A., Barrera G., Caputo E., Dianzani C., Pizzimenti S. (2023). Nrf2 as a Therapeutic Target in the Resistance to Targeted Therapies in Melanoma. Antioxidants.

[40] Bellmann L., Cappellano G., Schachtl-Riess J.F., Prokopi A., Seretis A., Ortner D., Tripp C.H., Brinckerhoff C.E., Mullins D.W., Stoitzner P. (2020). A TLR7 agonist strengthens T and NK cell function during BRAF-targeted therapy in a preclinical melanoma model. Int. J. Cancer..

[41] Comunanza V., Gigliotti C., Lamba S., Doronzo G., Vallariello E., Martin V., Isella C., Medico E., Bardelli A., Sangiolo D. (2023). Dual VEGFA/BRAF targeting boosts PD-1 blockade in melanoma through GM-CSF-mediated infiltration of M1 macrophages. Mol. Oncol..

[42] Golden E.B., Frances D., Pellicciotta I., Demaria S., Helen Barcellos-Hoff M., Formenti S.C. (2014). Radiation fosters dose-dependent and chemotherapy-induced immunogenic cell death. Oncoimmunology.

[43] Zhai J., Gu X., Liu Y., Hu Y., Jiang Y., Zhang Z. (2023). Chemotherapeutic and targeted drugs-induced immunogenic cell death in cancer models and antitumor therapy: An update review. Front. Pharmacol..

[44] Terlikowska K.M., Dobrzycka B., Terlikowski S.J. (2024). Modifications of Nanobubble Therapy for Cancer Treatment. Int. J. Mol. Sci..

[45] Baroni S., Argenziano M., La Cava F., Soster M., Garello F., Lembo D., Cavalli R., Terreno E. (2023). Hard-Shelled Glycol Chitosan Nanoparticles for Dual MRI/US Detection of Drug Delivery/Release: A Proof-of-Concept Study. Nanomaterials.

[46] Argenziano M., Bessone F., Dianzani C., Cucci M.A., Grattarola M., Pizzimenti S., Cavalli R. (2022). Ultrasound-Responsive Nrf2-Targeting siRNA-Loaded Nanobubbles for Enhancing the Treatment of Melanoma. Pharmaceutics.

[47] Hashemi M., Zandieh M.A., Talebi Y., Rahmanian P., Shafiee S.S., Nejad M.M., Babaei R., Sadi F.H., Rajabi R., Abkenar Z.O. (2023). Paclitaxel and docetaxel resistance in prostate cancer: Molecular mechanisms and possible therapeutic strategies. Biomed. Pharmacother..

[48] Wu M., Wang Y., Wang Y., Zhang M., Luo Y., Tang J., Wang Z., Wang D., Hao L., Wang Z. (2017). Paclitaxel-loaded and A10-3.2 aptamer-targeted poly(lactide-co-glycolic acid) nanobubbles for ultrasound imaging and therapy of prostate cancer. Int. J. Nanomed..

[49] Lan M., Zhu L., Wang Y., Shen D., Fang K., Liu Y., Peng Y., Qiao B., Guo Y. (2020). Multifunctional nanobubbles carrying indocyanine green and paclitaxel for molecular imaging and the treatment of prostate cancer. J. Nanobiotechnology.

[50] Chan M.H., Chan Y.C., Liu R.S., Hsiao M. (2020). A selective drug delivery system based on phospholipid-type nanobubbles for lung cancer therapy. Nanomedicine.

[51] Wang J.P., Yan J.P., Xu J., Yin T.H., Zheng R.Q., Wang W. (2019). Paclitaxel-loaded nanobubble targeted to pro-gastrin-releasing peptide inhibits the growth of small cell lung cancer. Cancer Manag. Res..

[52] Zhong S., Ling Z., Zhou Z., He J., Ran H., Wang Z., Zhang Q., Song W., Zhang Y., Luo J. (2020). Herceptin-decorated paclitaxel-loaded poly(lactide-*co*-glycolide) nanobubbles: Ultrasound-facilitated release and targeted accumulation in breast cancers. Pharm. Dev. Technol..

[53] Gebhardt C., Simon S.C.S., Weber R., Gries M., Mun D.H., Reinhard R., Holland-Letz T., Umansky V., Utikal J. (2021). Potential therapeutic effect of low-dose paclitaxel in melanoma patients resistant to immune checkpoint blockade: A pilot study. Cell. Immunol..

[54] Jordan M.A., Wilson L. (2004). Microtubules as a target for anticancer drugs. Nat. Rev. Cancer..

